# Prevalence of type 1 and type 2 diabetes in children and adolescents in Germany from 2002 to 2020: A study based on electronic health record data from the DPV registry

**DOI:** 10.1111/1753-0407.13339

**Published:** 2022-12-14

**Authors:** Anna Stahl‐Pehe, Clemens Kamrath, Nicole Prinz, Thomas Kapellen, Ulrike Menzel, Olga Kordonouri, K. Otfried Schwab, Susanne Bechtold‐Dalla Pozza, Joachim Rosenbauer, Reinhard W. Holl

**Affiliations:** ^1^ German Diabetes Center, Institute for Biometrics and Epidemiology Leibniz Center for Diabetes Research at Heinrich Heine University Düsseldorf Germany; ^2^ German Center for Diabetes Research (DZD) München Germany; ^3^ Center of Child and Adolescent Medicine Justus Liebig University Giessen Germany; ^4^ Institute of Epidemiology and Medical Biometry, Central Institute for Biomedical Technology (ZIBMT) Ulm University Ulm Germany; ^5^ Hospital for Children and Adolescents “Am Nicolausholz” Bad Kösen Bad Kösen Germany; ^6^ Department of Pediatric Endocrinology AKK Altonaer Kinderkrankenhaus Hamburg Germany; ^7^ Children's Hospital AUF DER BULT, Hannover Medical School Hannover Germany; ^8^ Department of Pediatrics and Adolescent Medicine, Pediatric Endocrinology, Diabetology and Lipidology, Faculty of Medicine University of Freiburg Freiburg Germany; ^9^ Pediatric Endocrinology and Diabetology, Dr. von Haunersches Kinderspital Ludwig‐Maximilians Medical University Munich Munich Germany

**Keywords:** epidemiology, prevalence, temporal trend, type 1 diabetes, type 2 diabetes, 流行病学, 1型糖尿病, 2型糖尿病, 患病率, 时间趋势

## Abstract

**Background:**

To provide estimates of the nationwide prevalence of type 1 diabetes (T1D) and type 2 diabetes (T2D) in individuals younger than 20 years of age in Germany from 2002 to 2020 and to identify trends.

**Methods:**

Data were obtained from the electronic health record “Diabetes Prospective Follow‐up Registry (DPV)” specific to diabetes care. Prevalence was estimated based on prevalent cases at the end of each year for the years 2002, 2008, 2014, and 2020 per 100 000 persons assuming a Poisson distribution and directly age‐ and/or sex‐standardized to the population in 2020. Individuals younger than 20 years of age with a clinical diagnosis of T1D or 10–19‐year‐olds with T2D were eligible for inclusion in the study.

**Results:**

The standardized T1D prevalence per 100 000 persons was 138.9 (95% CI: 137.1; 140.6) in 2002 and 245.6 (243.1; 248.0) in 2020. The standardized T2D prevalence per 100 000 persons was 3.4 (3.1; 3.8) in 2002 and 10.8 (10.1; 11.5) in 2020. The annual percent change (APC) in prevalence declined over the three periods 2002–2008/2008–2014/2014–2020 (T1D: 6.3% [3.6%; 9.0%]/3.1% [0.7%; 5.5%]/0.5% [−1.7%; 2.85], T2D: 12.3% [5.3%; 20.8%]/4.7% [−0.6%; 10.3%]/3.0% [−1.8%; 8.0%]). From 2014 to 2020, the highest APCs were observed among 15–19‐year‐olds (T1D: 2.5% [1.3%; 3.6%], T2D: 3.4% [−0.5%; 7.5%]).

**Conclusions:**

The increase in diabetes prevalence has slowed, but medical care should be prepared for an increase in adolescents with diabetes.

## Graphical abstract

**Highlights**

- The prevalence rates of type 1 diabetes and type 2 diabetes in Germany are higher than estimated by the International Diabetes Federation (IDF).
- Since 2014, prevalence rates have increased only in 15–19‐year‐olds.

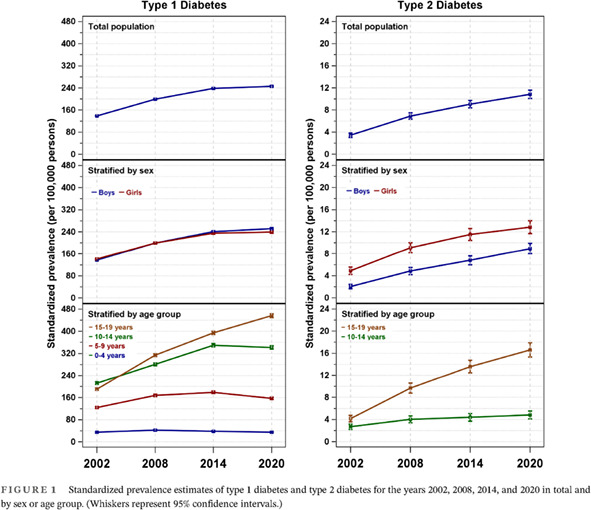

## 摘要

研究背景:评估2002~2020年德国20岁以下人群中1型糖尿病(T1D)和2型糖尿病(T2D)的患病率, 并确定其趋势。

方法:数据来源于糖尿病前瞻性随访登记系统(Diabetes Prospective Follow‐up Registry, DPV)。根据2002年、2008年、2014年和2020年每10万人年末发病例数, 采用Poisson分布和直接对2020年人群进行年龄和(或)性别标化, 估算患病率。小于20岁临床诊断为T1D的患者或10~19岁的T2D患者符合纳入标准。

结果:在2020年, 标化T1D患病率为138.9 / 10万(95% CI: 137.1;140.6)和245.6 (243.1;248.0), 标化T2D患病率为3.4/10万 (3.1;3.8)和10.8 (10.1;11.5)。2002‐2008年/ 2008‐2014年/ 2014‐2020年3个时期患病率的年度变化百分比(APC)均呈下降趋势(T1D: 6.3% (3.6%;9.0%) / 3.1% (0.7%;5.5%) / 0.5% (‐1.7%;2.85)、T2d:12.3% (5.3%;20.8%) / 4.7% (‐0.6%;10.3%) / 3.0% (‐1.8%;8.0%))。2014‐2020年, 15 ~ 19岁人群APC最高(T1D: 2.5% (1.3%;3.6%), T2D: 3.4% (‐0.5%;7.5%))。

结论:青少年糖尿病患病率的增长速度有所减缓, 但应做好医疗保健准备。

## 1 BACKGROUND

Worldwide, 1.5 million children and adolescents under 20 years of age have type 1 diabetes.^1^ In addition, there is currently an unknown number of children with type 2 diabetes and other types of diabetes because of the paucity of studies reporting on these diabetes types in adolescence.^2^ Observations from Western countries suggest an increase in the prevalence rates of type 1 diabetes^3^, ^4^, ^5^, ^6^, ^7^, ^8^, ^9^ and type 2 diabetes,^7^, ^8^ and these rates vary in different population groups. However, stagnating trends have also been reported from Europe for type 1 diabetes^4^ and type 2 diabetes.^9^, ^10^

Germany is listed among the 10 countries or International Diabetes Federation (IDF) areas with the highest estimated number of children and adolescents with type 1 diabetes. According to the most recent IDF atlas, there were 35.1 thousand prevalent cases of type 1 diabetes in children and adolescents in Germany in 2021.^11^ According to an estimation by Gregory et al., there were 40.2 (38.8–41.6) thousand people aged 0–19 years in Germany living with type 1 diabetes in 2021.^1^ For type 2 diabetes, the estimated prevalence in Germany is in the range of the European region (0.6–2.7 per 100 000 children and adolescents).^11^ However, a current and comprehensive Germany‐wide analysis of diabetes prevalence during childhood and adolescence is not available. Rather, the IDF estimates were derived by modeling the relationship between prevalence, incidence, onset age, and disease duration.^3^, ^12^ The references cited are based on data from no more than three regional registries in Germany collected through 2014.^13^, ^14^ The study by Gregory et al. used the IDF atlas and additional predictors.^1^ Further nationwide studies are required to improve the accuracy of the estimates.

The most recently published nationwide prevalence estimates for Germany were based on data from 2008 for 0–14‐year‐olds with type 1 diabetes^15^ and on data from 2014 to 2016 for 11–18‐year‐olds with type 2 diabetes.^16^ In addition, there are estimates for individual German federal states (type 1 diabetes prevalence among 0–14‐year‐olds in Saxony in 1999–2019,^17^ type 1 diabetes prevalence among 0–19‐year‐olds in North Rhine‐Westphalia in 2010^18^ and in 2002–2020,^19^ type 2 diabetes prevalence among 0–19‐year‐olds in Baden‐Württemberg in 2004/2005 and 2016,^10^ and type 2 diabetes prevalence among 10–19‐year‐olds in North Rhine‐Westphalia in 2002–2020^19^). Since the regional prevalence of diabetes is unevenly distributed, it is not possible to determine the Germany‐wide prevalence from single‐region data without restrictions.^20^

Knowing the current prevalence of type 1 diabetes and type 2 diabetes at the population level is a prerequisite for the establishment and further development of the diabetes care infrastructure. In addition, health surveillance, including trend analysis, has the potential to identify risk factors and determine the impact of preventive measures. The aim of this study was therefore to provide nationwide estimates of the prevalence of type 1 diabetes and type 2 diabetes in children and adolescents younger than 20 years of age in Germany for the period 2002–2020 and to identify trends.

The research question was whether the prevalence of type 1 diabetes and type 2 diabetes in children and adolescents changed in Germany from 2002 to 2008, 2014 and 2020 both overall and stratified by sex and age groups.

## 2 METHODS

This study followed the Strengthening the Reporting of Observational Studies in Epidemiology (STROBE) guideline for cohort studies^21^ and the Reporting of Studies Conducted Using Observational Routinely Collected Data (RECORD) statement.^22^

### 2.1 Database

For this study, we used data from the multinational Diabetes Prospective Follow‐up Registry (Diabetes‐Patienten‐Verlaufsdokumentation, DPV). The DPV diabetes documentation system is an electronic health record specific for standardized documentation of diabetes care that has been used extensively for research and the assessment of the quality of healthcare delivery.^23^ Twice a year, the locally collected longitudinal data are pseudonymized and transmitted to the University of Ulm, Ulm, Germany, for central plausibility checks and analyses, including screening for erroneous diagnoses, erroneous and missing data, and duplicate records. Inconsistent data are reported back to the participating centers for validation and/or correction. The data are then completely anonymized for analysis. Based on data from North Rhine‐Westphalia, the most populous state in Germany, an updated analysis estimated DPV completeness for the period 2002–2020. According to these estimations, the DPV contained, on average, records of 93% of pediatric patients with type 1 diabetes and records of 72% of pediatric patients with type 2 diabetes.^16^, ^18^ As of November 11, 2021, 325 (175) clinics and practices in Germany registered at least one patient with type 1 diabetes (type 2 diabetes) in the DPV for 2020. The centers and clinics are responsible for obtaining verbal or written informed consent for participation in the DPV from patients or their guardians to maintain data privacy. The ethics committee of the University of Ulm, Ulm, Germany, approved the analysis of the anonymized DPV data (no. 314/21).^23^, ^24^

### 2.2 Study population selection

Patients with a clinical diagnosis of type 1 diabetes under the age of 20 years and patients with a clinical diagnosis of type 2 diabetes between 10 and < 20 years of age as of December 31 in 2002, 2008, 2014, and 2020 were eligible for inclusion in the study. The lower age limit for type 2 diabetes patients was selected as 10 years because the case numbers for younger individuals would be too small for valid prevalence estimates.^7^ The last date of registration in the dataset was September 15, 2021.

Diabetes type was initially determined by the treating physicians at the onset of diabetes and, if necessary, revised at the annual data update based on additional clinical and laboratory data in the course of the disease, according to national guidelines,^25^ which are consistent with International Society for Pediatric and Adolescent Diabetes (ISPAD) diagnostic criteria.^26^ Population data were obtained from the Federal Statistical State Office of Germany.^27^

### 2.3 Statistical analysis

Prevalence rates were estimated based on prevalent cases on December 31 in 2002, 2008, 2014, and 2020 and are reported per 100 000 persons. Prevalence rates were obtained by dividing the numbers of registered persons by annual population estimates. Point and interval estimates (corresponding 95% CIs) of prevalence (per 100 000 persons) were based on the Poisson distribution. We provide crude and standardized total and interval estimates (95% CI) for each of the two types of diabetes as well as sex‐ and age‐specific prevalence rates (age groups 0–4, 5–9, 10–14, and 15–19 years for type 1 diabetes and type 2 diabetes; 10–14 and 15–19 years for type 2 diabetes). Standardization of rates was performed by the direct method with the age and sex distribution of the 2020 population as the standard population. In addition, differences and the annual percent change (APC) assuming a log‐linear trend within the respective period between prevalence estimates at the end and beginning of each of the 6‐year intervals (2002–2008, 2008–2014, and 2014–2020) were estimated from Poisson models. Poisson models accounted for overdispersion by inflating the variance estimations with the factor “deviance/degrees of freedom” (quasi‐likelihood estimation method).^28^ For the total group, point and interval estimates of prevalence differences and APCs were calculated from the regression coefficients and corresponding standard errors of Poisson regression models including year, sex, and age group as categorical terms. For age‐ or sex‐specific estimates, an age‐by‐year or sex‐by‐year interaction term was added to the Poisson model. Accordingly, the APC was estimated for the 18‐year period (2002–2020) by modeling year as a continuous term. CIs of prevalence differences and APCs were adjusted for multiple inference according to the Bonferroni method separately within each stratum (total group, boys, girls, age groups). Within the respective Poisson model, the equality of the period‐specific APCs (2002–2008, 2008–2014, and 2014–2020) was assessed, and thus, a unique log‐linear trend over the total period 2002–2020 was tested by a likelihood ratio test. A statistically significant *p* value (*p* value <0.05) indicated differences in the period‐specific APCs and thus deviations from a uniform overall log‐linear trend. All analyses were performed using SAS version 9.4 (2016; SAS Institute Inc., Cary, North Carolina).

## 3 RESULTS

### 3.1 Type 1 diabetes

**FIGURE 1:**
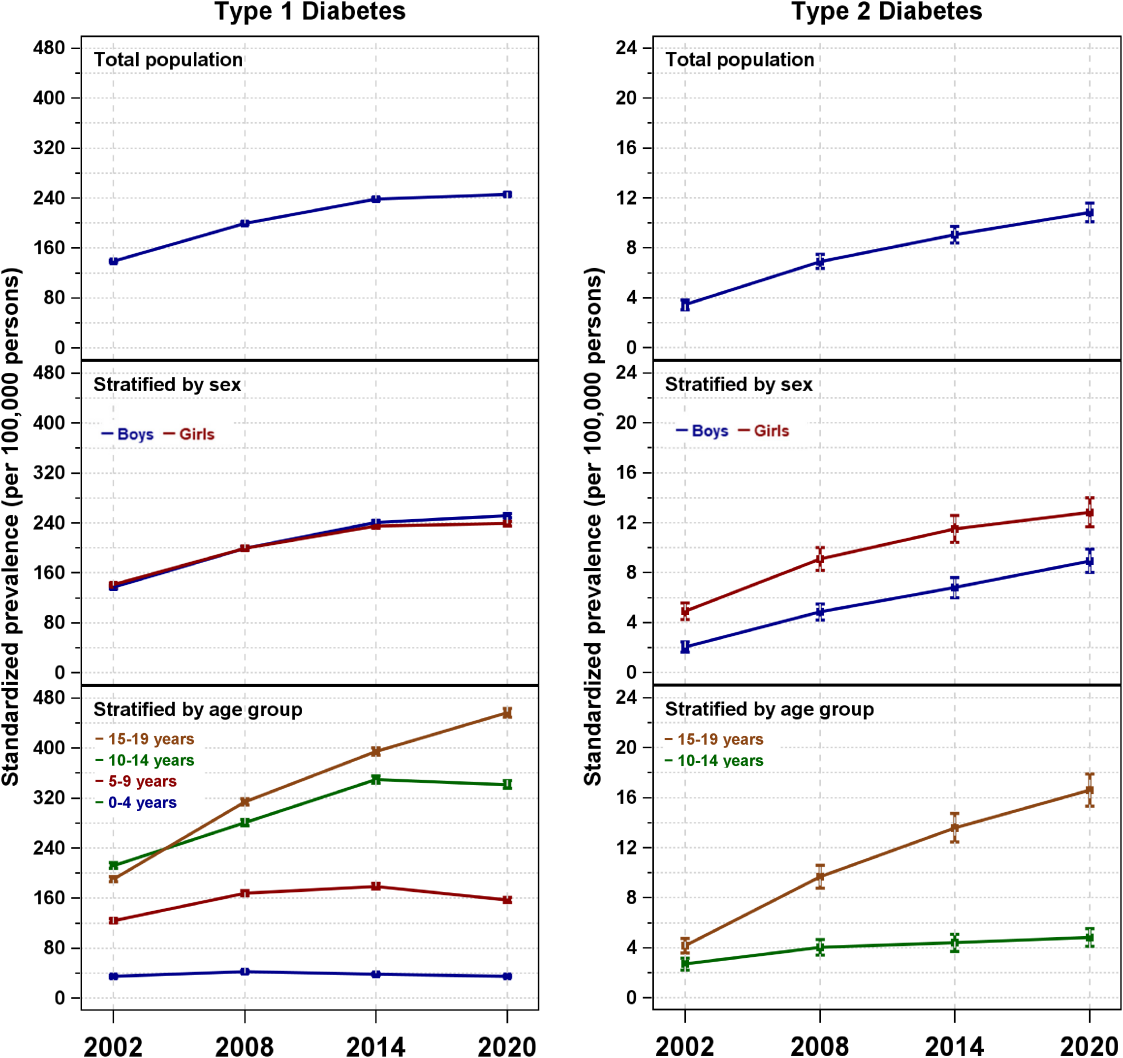
Standardized prevalence estimates of type 1 diabetes and type 2 diabetes for the years 2002, 2008, 2014, and 2020 in total and by sex or age group. (Whiskers represent 95% confidence intervals.)

Table 1 shows that a total of 24.9 thousand individuals younger than 20 years of age with type 1 diabetes were identified from a population of 17.1 million individuals in this age range at the end of 2002, corresponding to a crude prevalence of 145.9 (95% CI: 144.1; 147.7) cases per 100 000 persons. The total number of prevalent cases and the crude prevalence increased in 2008, 2014, and 2020, mainly due to increases in the oldest age group. At the end of 2020, a total of approximately 37.7 thousand individuals with type 1 diabetes were identified from a population of 15.3 million individuals, corresponding to a crude prevalence of 245.6 (243.1; 248.0) cases per 100 000 persons. For 2002–2014, the age‐ and sex‐standardized prevalence was estimated to be lower than the crude prevalence. More boys than girls with type 1 diabetes were recorded in the DPV at all survey time points. At the first three survey time points, the crude and age‐standardized prevalence was similar for girls and boys; however, at the last time point, the standardized type 1 diabetes prevalence was higher for boys than for girls, as the nonoverlapping CIs indicate. At most survey time points, an increase in the standardized prevalence with increasing age was apparent (Table 1, Figure 1).

**TABLE 1:** Crude and standardized prevalence of type 1 diabetes and type 2 diabetes in 2002, 2008, 2014, and 2020

	2002		2008		2014		2020	
	No. of cases/total population	Prevalence per 100 000 persons (95% CI)	No. of cases/total population	Prevalence per 100 000 persons (95% CI)	No. of cases/total population	Prevalence per 100 000 persons (95% CI)	No. of cases/total population	Prevalence per 100 000 persons (95% CI)
		Crude		Crude		Crude		Crude
Characteristic		Standardized		Standardized		Standardized		Standardized
Type 1 diabetes								
Total	24 933/17 089 016	145.9 (144.1; 147.7)	32 915/15 618 736	210.7 (208.5; 213.0)	36 577/14 753 511	247.9 (245.4; 250.5)	37 655/15 334 574	245.6 (243.1; 248.0)
		138.9 (137.1; 140.6)		199.5 (197.3; 201.6)		238.1 (235.7; 240.6)		245.6 (243.1; 248.0)
Sex								
Boys	12 609/8 769 723	143.8 (141.3; 146.3)	16 899/8 010 280	211.0 (207.8; 214.2)	19 014/7 578 301	250.9 (247.4; 254.5)	19 896/7 887 852	252.2 (248.7; 255.8)
		136.9 (134.5; 139.2)		199.5 (196.5; 202.6)		240.6 (237.2; 244.1)		251.8 (248.3; 255.3)
Girls	12 324/8 319 293	148.1 (145.5; 150.8)	16 016/7 608 456	210.5 (207.3; 213.8)	17 563/7 175 210	244.8 (241.2; 248.4)	17 759/7 446 722	238.5 (235.0; 242.0)
		140.9 (138.4; 143.4)		199.4 (196.3; 202.5)		235.4 (231.9; 238.9)		238.9 (235.4; 242.4)
Age, years								
0–4	1 316/3 804 521	34.6 (32.8; 36.5)	1 448/3 445 172	42.0 (39.9; 44.3)	1 332/3 486 411	38.2 (36.2; 40.3)	1 390/3 969 138	35.0 (33.2; 36.9)
		34.6 (32.7; 36.5)		42.0 (39.9; 44.2)		38.2 (36.2; 40.3)		35.0 (33.2; 36.9)
5–9	4 963/4 005 841	123.9 (120.5; 127.4)	6 246/3 714 997	168.1 (164.0; 172.4)	6 242/3 491 478	178.8 (174.4; 183.3)	5 934/3 783 568	156.8 (152.9; 160.9)
		123.9 (120.4; 127.3)		168.1 (164.0; 172.3)		178.8 (174.4; 183.2)		156.8 (152.8; 160.8)
10–14	9 771/4 605 217	212.2 (208.0; 216.4)	11 166/3 978 937	280.6 (275.5; 285.9)	12 960/3 708 834	349.4 (343.5; 355.5)	12 719/3 725 094	341.7 (335.8; 347.7)
		212.2 (208.0; 216.4)		280.6 (275.4; 285.8)		349.4 (343.4; 355.4)		341.7 (335.8; 347.7)
15–19	8 883/4 673 436	190.1 (186.1; 194.0)	14 055/4 479 630	313.8 (308.6; 318.9)	16 043/4 066 788	394.5 (388.4; 400.6)	17 602/3 856 774	456.4 (449.7; 463.2)
		190.1 (186.1; 194.0)		313.8 (308.6; 319.0)		394.5 (388.4; 400.6)		456.3 (449.6; 463.1)
Type 2 diabetes								
Total	318/9 278 653	3.4 (3.1; 3.8)	595/8 458 567	7.0 (6.5; 7.6)	716/7 775 622	9.2 (8.6; 9.9)	819/7 581 868	10.8 (10.1; 11.6)
		3.4 (3.1; 3.8)		6.9 (6.4; 7.5)		9.1 (8.4; 9.7)		10.8 (10.1; 11.6)
Sex								
Boys	97/4 760 188	2.0 (1.7; 2.5)	213/4 337 563	4.9 (4.3; 5.6)	276/3 998 397	6.9 (6.1; 7.8)	350/3 907 597	9.0 (8.0; 10.0)
		2.0 (1.6; 2.5)		4.8 (4.2; 5.5)		6.8 (6.0; 7.6)		9.0 (8.0; 9.9)
Girls	221/4 518 465	4.9 (4.3; 5.6)	382/4 121 004	9.3 (8.4; 10.3)	440/3 777 225	11.7 (10.6; 12.8)	469/3 674 271	12.8 (11.7; 14.0)
		4.9 (4.3; 5.6)		9.1 (8.2; 10.0)		11.5 (10.4; 12.6)		12.8 (11.7; 14.0)
Age, years								
10–14	124/4 605217	2.7 (2.2; 3.2)	161/3 989937	4.1 (3.5; 4.7)	163/3708 834	4.4 (3.8; 5.1)	180/3 725 094	4.8 (4.2; 5.6)
		2.7 (2.2; 3.2)		4.0 (3.4; 4.7)		4.4 (3.7; 5.1)		4.8 (4.1; 5.5)
15–19	194/4 673 436	4.2 (3.6; 4.8)	434/4 479 630	9.7 (8.8; 10.6)	553/4 066 788	13.6 (12.5; 14.8)	639/3 856 774	16.6 (15.4; 18.0)
		4.1 (3.6; 4.7)		9.7 (8.8; 10.6)		13.6 (12.5; 14.7)		16.6 (15.3; 17.9)

When comparing the model‐based estimates of differences and the APCs of the three periods given in Table 2, it is noticeable that the APCs decreased from one period to the next, both overall and in groups stratified by sex. The total type 1 diabetes prevalence increased annually by 6.3% (3.6%; 9.0%) from 2002 to 2008, 3.1% (0.7%; 5.5%) from 2008 to 2014, and 0.5% (−1.7%; 2.8%) from 2014 to 2020, resulting in an overall annual 3.1% (2.3%; 3.9%) increase over the entire period. Differentiated by age groups, the results are diverse. Only in the highest age group did prevalence increase in all three periods. However, the prevalence increase declined over time (Figure 1). A uniform log‐linear trend across the three periods was not found except among 0–4‐year‐olds, as indicated by the *p* values shown in Table 2.

**TABLE 2:** Model‐based difference and annual percent change in the prevalence of type 1 diabetes and type 2 diabetes among youths in 2002, 2008, 2014, and 2020

	Difference in prevalence per 100 000 youths (95% CI)^a^ ^,^ ^b^			Annual percent change in estimated prevalence (95% CI), %^a^ ^,^ ^b^				
Characteristic	2008 vs. 2002	2014 vs. 2008	2020 vs. 2014	2002–2008	2008–2014	2014–2020	*p* value for uniform log‐linear trend^c^	2002–2020
Type 1 diabetes								
Total	61.2 (35.5; 87.0)	40.1 (9.9; 70–6)	7.2 (−25.4; 39.9)	6.3 (3.6; 9.0)	3.1 (0.7; 5.5)	0.5 (−1.7; 2.8)	<0.001	3.1 (2.3; 3.9)
Sex								
Boys	63.5 (25.6; 101.4)	42.6 (−2.5; 87.8)	10.9 (−37.7; 59.5)	6.6 (2.6; 10.7)	3.3 (−0.2; 6.8)	0.7 (−2.5; 4.1)	0.026	3.3 (2.1; 4.5)
Girls	58.9 (19.8; 98.0)	37.5 (−8.5; 83.6)	3.3 (−45.8; 52.4)	6.0 (2.0; 10.1)	2.9 (−0.6; 6.6)	0.2 (−3.1; 3.7)	0.034	2.9 (1.7; 4.0)
Age, years								
0–4	7.4 (−1.5; 16.4)	−3.8 (−13.1; −5.5)	−3.2 (−11.8; 5.4)	3.3 (−0.6; 7.4)	−1.6 (−5.3; 2.3)	−1.4 (−5.2; 2.5)	0.077	−0.1 (−1.2; 1.0)
5–9	44.2 (27.3; 61.1)	10.6 (−8.4; 29.7)	−21.9 (−40–6; −3.3)	5.2 (3.2; 7.3)	1.0 (−0.8; 2.9)	−2.2 (−3.9; −0.3)	<0.001	1.2 (−0.2; 2.6)
10–14	68.4 (47.5; 89.3)	68.8 (43.9; 93.6)	−7.7 (−34.1; 18.7)	4.8 (3.3; 6.3)	3.7 (2.4; 5.1)	−0.4 (−1.6; 0.9)	<0.001	2.7 (1.6; 3.8)
15–19	123.7 (103.3; 144.0)	80.7 (55.7; 105.7)	61.9 (33.5; 90.3)	8.7 (7.2; 10.2)	3.9 (2.7; 5.1)	2.5 (1.3; 3.6)	<0.001	4.6 (3.4; 5.8)
Type 2 diabetes								
Total	3.5 (1.5; 5.4)	2.2 (0.0.3; 4.7)	1.7 (−1.1; 4.6)	12.3 (5.3; 19.8)	4.7 (−0.6; 10.3)	3.0 (−1.8; 8.0)	0.032	5.8 (4.1; 7.6)
Sex								
Boys	2.8 (0.6; 4.9)	2.0 (−1.0; 4.9)	2.1 (−1.4; 5.6)	15.3 (2.8; 29.4)	5.9 (−2.8; 15.4)	4.7 (−3.0; 12.9)	0.225	7.4 (4.7; 10.2)
Girls	4.1 (1.0; 7.3)	2.4 (−1.6; 6.4)	1.3 (−3.2; 5.8)	10.8 (2.3; 20.0)	4.0 (−2.6; 11.1)	1.8 (−4.3; 8.4)	0.129	4.9 (2.5; 7.3)
Age, years								
10–14	1.4 (−0.2; 3.0)	0.4 (−1.5; 2.2)	0.4 (−1.5; 2.4)	7.0 (−1.1; 15.8)	1.4 (−5.8; 9.1)	1.6 (−5.4; 9.1)	0.441	3.0 (1.4; 4.6)
15–19	5.6 (3.4; 7.8)	4.0 (1.0; 6.9)	3.1 (−0.4; 6.5)	15.2 (8.8; 21.9)	5.8 (1.5; 10.4)	3.4 (−0.5; 7.5)	<0.001	6.9 (4.4; 9.4)

^a^ Estimates are derived from Poisson models including categorical terms for year, sex, and age and a sex‐by‐year or age‐by‐year interaction term for sex‐ and age‐specific estimates, respectively, assuming a uniform trend within each period.

^b^ CIs are adjusted for multiple inference according to the Bonferroni method separately within each stratum.

^c^ The *p* value relates to a likelihood ratio test for testing the equality of the period‐specific annual percent changes and thus for a unique overall log‐linear trend within the respective Poisson model. A significant *p* value indicates differences between the period‐specific annual percent changes and thus deviations from a unique overall log‐linear trend.

### 3.2 Type 2 diabetes

The total crude type 2 diabetes prevalence in 10–19‐year‐olds was 3.4 (3.1; 3.8) per 100 000 persons at the end of 2002 (corresponding to 318 youths identified from a population of 9.3 million youths). There was an increase in the prevalence at subsequent time points, especially among 15–19‐year‐olds, as shown in Figure 1. The increase resulted in a type 2 diabetes prevalence of 10.8 (10.1; 11.6) per 100 000 persons at the end of 2020 (corresponding to 819 youths identified from a population of 7.6 million youths). The estimated standardized type 2 diabetes prevalence was 1.4 times higher among girls (12.8 [11.7; 14.0]) than boys (9.0 [8.0; 9.9]) and 3.4 times higher among older adolescents (16.6 [15.3; 17.9]) than younger adolescents (4.8 [4.1; 5.5]) in 2020 (Table 1).

The model‐based estimates of differences and APCs in estimated type 2 diabetes prevalence over time suggest that the increase in prevalence was higher in the first period than in subsequent periods, although CIs were wide. The total type 2 diabetes prevalence increased annually by 12.3% (5.3%; 19.8%) from 2002 to 2008, 4.7% (−0.6%; 10.3%) from 2008 to 2014, and 3.0% (−1.8%; 8.0%) from 2014 to 2020, resulting in an overall 5.8% (4.1%; 7.6%) annual increase during the period 2002–2020. The overall rise was more pronounced in boys and among 15–19‐year‐olds. A uniform log‐linear trend across the three periods was rejected among the total group and the 15–19‐year‐olds, as indicated by the *p* values shown in Table 2.

## 4 DISCUSSION

This study provides estimates of the prevalence rates of type 1 diabetes and type 2 diabetes in children and adolescents in Germany for the period 2002–2020 based on nationwide electronic health record data. The prevalence rates in 2020 were higher than 18 years ago due to an increase in the numbers of people with type 1 and type 2 diabetes on the one hand and a decrease in the population on the other hand. The numbers of prevalent cases with type 1 diabetes and type 2 diabetes were higher than the IDF estimates.^11^ Thus, the IDF extrapolations based on data from regional incidence registries resulted in an underestimation of the prevalence rates.

### 4.1 Comparison with previous studies

Our observations regarding type 1 diabetes prevalence in 2020 and over time show both similarities and differences with previous studies from Western countries.^4^, ^6^, ^7^, ^9^ Our estimated type 1 diabetes prevalence for 0–19‐year‐olds in 2020 was higher than that previously reported from the Netherlands,^4^ Hungary,^9^ the SEARCH for Diabetes in Youth (SEARCH) study in six regions of the USA,^7^ and Canada.^6^ Others have observed a higher prevalence in boys, which is similar to our findings.^6^, ^9^ The 3.1% APC for type 1 diabetes was similar to other countries (2.8% in Canada 2002–2013,^6^ 3.8% in the Netherlands 1998–2011,^4^ 5.8% in Hungary 2001–2016^9^). We observed that the type 1 diabetes prevalence increase declined during the observation period. A similar trend was also reported from US areas, where the average APC was 3.4% in 2001–2009 and 1.4% in 2009–2017.^7^ The APC was the same for girls and boys in our study as in other surveys.^4^, ^7^ Similar to the neighboring country the Netherlands,^4^ Germany has had a negative APC in the youngest age group since 2008 and the highest APC in the oldest age group. In the past, a shift to an earlier clinical manifestation of type 1 diabetes had been reported.^29^, ^30^, ^31^ Our update, however, shows that the prevalence in young children remains unchanged. Overall, there are indications that the type 1 diabetes incidence increase slows down, and thus, a prevalence plateau has been reached, as previously reported based on data from 0–14‐year‐olds from the German federal state Saxony.^17^

Classification of our results regarding type 2 diabetes based on comparisons with other studies is limited due to a small number of studies reporting on type 2 diabetes in adolescence.^2^ In addition, sometimes large methodological differences prevent a meaningful comparison (e.g., estimates not stratified by age groups^9^, ^10^). A systematic review reported a wide range of 0–5300 per 100 000 children and adolescents for type 2 diabetes prevalence due to both population characteristics and methodological differences.^32^ We therefore compare our results only with studies conducted in Germany or other Western countries during a comparable period. In 2016, the prevalence of type 2 diabetes among 11–18‐year‐olds in Germany was estimated using the North Rhine‐Westphalia Diabetes Registry, prevalence data from Baden‐Württemberg, and the DPV database. The estimated prevalence, adjusted for underreporting, was 12.2 per 100 000 among 11–18‐year‐olds and was 1.6 times higher in girls than in boys and 2.5 times higher in 15–18‐year‐olds than in 11–14‐year‐olds.^16^ Our estimated prevalence was slightly lower than that in the aforementioned study. The difference between sexes was similar, but the difference between age groups was significantly larger. In the SEARCH study, the prevalence was 67 per 100.000 10–19‐year‐olds, which was several times higher than in Germany (data from 10–19‐year‐olds, 2017). However, the preponderance of girls (1.6 times higher prevalence in girls than in boys) and older adolescents (3.6 times higher prevalence in 15–19‐year‐olds than in 10–14‐year‐olds) was similar. An increase in type 2 diabetes prevalence was observed in both our data and US data. While in the US areas the APC increased linearly by 3.7% in 2001–2009 and by 4.8% in 2009–2017, a weakening increase was seen in our data. The highest APCs were observed in non‐Hispanic Black and Hispanic adolescents, while only a slight increase was observed among White adolescents.^7^ We suspect, as do other authors, that the difference in prevalence and trends is not only due to methodological reasons (e.g., screening methods) but can also be explained by ethnic differences, differences in life circumstances (e.g., exposure to maternal obesity and diabetes, obesity‐promoting environment), and the prevalence of anthropometric risk factors (e.g., body mass index).^7^, ^8^, ^16^, ^32^, ^33^

### 4.2 Strengths and limitations

The strengths of this study include the long study period during which a consistent methodology was used and the assessment of diabetes type by treating physicians. The methodology was similar to that of the SEARCH study^7^ but was different from that used in other studies in which prevalence was determined using prescriptions of antidiabetic treatment^4^, ^9^ or a combination of datasets.^20^ Due to the use of the DPV database, the risk of misclassification and changing eligibility over time was low. Because data from all DPV centers were used and no selection was performed, the risks of information and selection bias are low. A further strength is that rates were adjusted for age and sex distribution of the population using official data, reducing bias in the comparison of prevalence rates. Finally, our data are suitable for use in the context of international research initiatives (e.g., European Diabetes [EURODIAB] register^14^ and IDF^3^) because we used common stratifications (girls/boys, 5‐year age groups) and methods of analysis (Poisson regression models).

A limitation is that the diabetes prevalence data reported here represent a conservative estimate for two reasons related to missing data. First, only adolescents with diagnosed diabetes were included. Undiagnosed cases were unlikely in adolescents with type 1 diabetes because of the severity of symptoms at baseline. In adolescents with type 2 diabetes, however, a relevant rate of undiagnosed cases in the early 2000s cannot be excluded. According to a systematic review, the reported prevalence of undiagnosed type 2 diabetes in 12–19‐year‐olds was 2.2% or less worldwide in 2010–2017.^34^ Second, the completeness of DPV coverage for all of Germany at the four data collection points is not known, so no estimate corrected for completeness can be provided. Despite likely undercoverage in type 2 diabetes, our estimated type 2 diabetes APC for 2002–2020 (5.8 [4.1; 7.6]) was similar to the underreporting‐adjusted APC in North Rhine‐Westphalia for the same period (6.1% [5.3%; 6.9%]).^19^ In addition, our estimates may show some overestimation bias. Due to the data collection procedure and data protection reasons, duplicate reports cannot be completely ruled out and may lead to an overestimation if, for example, a patient moves and does not report the same manifestation date. In addition, a systematic follow‐up regrading deaths and emigration has not been implemented in the DPV. However, since mortality in young people with diabetes in Western countries is very low,^35^ the results should not be seriously affected by mortality. Overall, we assume, based on updated analyses regarding registration coverage,^16^, ^18^ that the actual prevalence of diagnosed type 1 diabetes and type 2 diabetes may be 10% and 30% higher than reported, respectively. Finally, our study lacked data to differentiate by region, living conditions, and ethnicity. Therefore, we cannot determine whether changes in population characteristics accounted for the differences observed over time.

Recently, there has been increasing evidence that the COVID‐19 pandemic is impacting diabetes incidence.^36^, ^37^, ^38^, ^39^, ^40^ We examined diabetes prevalence in four calendar years, each 6 years apart, and were unable to draw conclusions about the association of COVID‐19 and diabetes prevalence based on our data. It will be the task of future research to follow further epidemiological developments and to investigate possible disease associations.

### 4.3 Implications

Our study may be of practical use in expanding knowledge of the epidemiology of type 1 diabetes and type 2 diabetes. At the national level, our data can be used to plan for health service needs and adjust resource allocation to address the continued increase in adolescent diabetes. Globally, this analysis contributes to an updated picture of diabetes prevalence in childhood and adolescence and provides valuable insights, particularly with regard to the scarce epidemiological research on type 2 diabetes in adolescence.

## 5 CONCLUSIONS

Over the past 20 years, the prevalence of both type 1 diabetes and type 2 diabetes in Germany has continued to increase, but the overall trends have leveled off over the years. This finding can be explained by the fact that the increase in prevalence continued among older adolescents, but the prevalence remained unchanged or decreased among the youngest children. Monitoring further development is necessary, especially in light of the emerging changes in the incidence patterns of type 1 diabetes and type 2 diabetes during the COVID‐19 pandemic.

## AUTHOR CONTRIBUTIONS

**Reinhard W. Holl:** Data acquisition. **Joachim Rosenbauer:** Statistical analyses. **Anna Stahl‐Pehe:** Writing – first draft of the manuscript. All authors commented on previous versions of the manuscript and read and approved the final version.

## CONFLICT OF INTEREST

None.

## FUNDING INFORMATION

The German Diabetes Center receives institutional funding from the German undercoverage Federal Ministry of Health (BMG) and the Ministry of Culture and Science of the State of North Rhine‐Westphalia (MKW NRW). The Diabetes‐Patienten‐Verlaufsdokumentation (DPV) is supported by the German Federal Ministry for Education and Research within the German Center for Diabetes Research (grant no. 82DZD14E03), the Robert Koch Institute (RKI), and the German Diabetes Association (DDG). The funding organizations had no role in the design and conduct of the study; collection, management, analysis, and interpretation of the data; preparation, review, or approval of the article; or the decision to submit the article for publication.

## DISCLOSURE

None.

## PATIENT CONSENT

Informed consent was obtained from participants or their parents/legal guardians.

## MEETING PRESENTATION

A subset of these data regarding type 1 diabetes was presented at the German Diabetes Association's (Deutsche Diabetes Gesellschaft, DDG) Congress on May 25, 2022.

## Data Availability

Access to aggregated data is possible after reasonable request.
